# On Neuroeducation: Why and How to Improve Neuroscientific Literacy in Educational Professionals

**DOI:** 10.3389/fpsyg.2021.752151

**Published:** 2021-12-03

**Authors:** Jelle Jolles, Dietsje D. Jolles

**Affiliations:** ^1^Faculty of Behavioural and Movement Sciences, Vrije Universiteit Amsterdam, Amsterdam, Netherlands; ^2^Leiden Institute for Brain and Cognition, Institute of Education and Child Studies, Faculty of Social and Behavioural Sciences, Leiden University, Leiden, Netherlands

**Keywords:** neuroeducation, brain development, executive functions, neuroscientific literacy, education, adolescence, brain plasticity, neuromyth

## Abstract

New findings from the neurosciences receive much interest for use in the applied field of education. For the past 15 years, neuroeducation and the application of neuroscience knowledge were seen to have promise, but there is presently some lack of progress. The present paper states that this is due to several factors. Neuromyths are still prevalent, and there is a confusion of tongues between the many neurodisciplines and the domains of behavioral and educational sciences. Second, a focus upon cognitive neuroimaging research has yielded findings that are scientifically relevant, but cannot be used for direct application in the classroom. A third factor pertains to the emphasis which has been on didactics and teaching, whereas the promise of neuroeducation for the teacher may lie more on pedagogical inspiration and support. This article states that the most important knowledge and insights have to do with the notion of *brain plasticity*; the vision that development is driven by an interaction between a person’s biology and the social system. This helps individuals to select and process information, and to adapt to the personal environment. The paper describes how brain maturation and neuropsychological development extend through the important period of adolescence and emergent adulthood. Over this long period, there is a major development of the Executive Functions (EFs) that are essential for both cognitive learning, social behavior and emotional processing and, eventually, personal growth. The paper describes the basic neuroscience knowledge and insights – or “neuroscientific literacy” – that the educational professional should have to understand and appreciate the above-described themes. The authors formulate a proposal for four themes of neuroscience content “that every teacher should know.” These four themes are based on the Neuroscience Core Concepts formulated by the Society for Neuroscience. The authors emphasize that integrating neuroscientific knowledge and insights in the field of education should not be a one-way street; attempts directed at improving neuroscientific literacy are a transdisciplinary undertaking. Teacher trainers, experts from the neuroscience fields but also behavioral scientists from applied fields (notable applied neuropsychologists) should all contribute to for the educational innovations needed.

## Introduction

In the past two decades there has been a rapid rise in the interest in research findings about the brain, especially in relation to learning, human cognition, and behavior. Advancing research methods have improved our understanding of the mechanisms underlying the way we learn, think, reason, and feel, from the perspective of the functioning of the human brain. Furthermore, researchers are improving our insights into the maturation of the brain and its relation to developmental changes in cognition, emotional functioning, and behavior (e.g., Mayer, 2017). The relevance of these findings for the domain of education is expressed in new books (e.g., Tokuhama-Espinosa, 2014; Blakemore, 2018; Steinberg, 2019; Dehaene, 2020), in much visited meetings such as the Learning and the Brain conferences in the United States, and the increasing interest in the topic of Neuroeducation, and in the establishment of new journals such as “Mind, Brain and Education” and “Trends in Neuroscience and Education.” These and other journals are important in encouraging the crosstalk between the multidimensional domains of neuroscience, behavioral and cognitive science, and the field of education (Ansari et al., 2017; Thomas et al., 2019).

Since the Decade of the Brain in the 1990ies (Jones and Mendell, 1999) and especially the OECD report on “the birth of a learning science” (OECD, 2007), there is also a rise in the interest of educators and policy makers for issues related to brain and education. Yet there is still some hesitation and even resistance to the notion that insights from neuroscience could ever be applied in the classroom, and some education researchers remain suspicious of what they regard as “a hype” surrounding educational neuroscience (e.g., Bowers, 2016; see Thomas et al., 2020 for an elaboration). Hence, the translation of neuroscience knowledge to the field of education and its successful application in educational settings is not at all settled. A major stumbling block in this translation is that educators who are really interested in applicable knowledge from the neurosciences have trouble finding the right books, articles and trustable internet sites. Moreover, university courses or programs integrating insights from the fields of neuroscience and education are still rare and not within reach of the majority of educators. The difficulty of finding reliable, accessible and relevant knowledge may be one of the reasons that many educational professionals believe in the so-called “neuromyths” (OECD, 2007; Dekker et al., 2012; Howard-Jones, 2014; Macdonald et al., 2017).

In terms of the metaphor used by Bruer (1997) and others (e.g., Ansari et al., 2017; Goswami, 2019): there are not enough bridges over the river that separates the field of education from the domain of the neurosciences, and the bridges that exists are not easily accessible to educators. Even among scientists in the field of Mind, Brain, and Education, there is no consensus as to which knowledge and insights about brain structure and function could be relevant for use in the domain of education, and which knowledge and insights is not. Therefore, it is the purpose of the present article to contribute in this respect. We defend the position that knowledge about the brain does not always have a *direct* relevance for applied fields, yet everybody should be familiar with the basic facts about brain structure and function, as well as its development, analogous to the common knowledge we have about the heart, about digestive function and the respiratory system.

With respect to the term “neuroscientific literacy” we use in this paper, we employ a definition which is based upon the earlier definitions proposed by Herculano-Houzel (2002) and others (e.g., Horvath et al., 2018; Im et al., 2018). Howard-Jones and coworkers (e.g., Howard-Jones, 2010; Deligiannidi and Howard-Jones, 2015) defined neuroscience literacy – or “neuroliteracy” – in terms of “understanding about the brain and how it functions.” Neuroscience literacy as we see it, can be defined as “*the knowledge and understanding of brain systems and processes required for cognitive and affective functioning across the lifespan, including neuroscience issues related to disease, disorders, and dysfunction, as well as notions how humans interact with their environment and with each other because of their nervous system characteristics*.” Note that it has been suggested to extend the notion of neuroscientific literacy, in order to incorporate “*the adoption of a critical-reflective teaching method*” (e.g., Bergmann et al., 2017). We appreciate the relevance of such an extension, but will use the former definition for the present article because of the earlier literature which used a similar definition. We propose that it is essential to improve neuroscientific literacy in terms of the educator’s understanding of the knowledge base that exists about brain structure and function. In addition, educators should be given the tools to decide what scientific evidence is valid and how to judge its quality. This is essential to allow them to reflect on the potential applicability. Experts in the field state that such insights can support teachers’ professional judgment and give them a better understanding of their students (Ansari et al., 2017; Goswami, 2019; see also Sigman et al., 2014). This will impact their pedagogical knowledge and give additional weight to their educational approach.

The present paper intends to be a perspective article which presents a viewpoint on “the new science of learning” (OECD, 2007) which is not only based upon insights from cognitive science, behavioral science and educational science but also on knowledge obtained in the neurosciences including neuropsychology. Importantly, this paper is *not* meant to provide neuroscientific support for specific kinds of educational practices, or to give concrete advice that is directly applicable in the classroom. Rather, it aims to provide an overview of relevant neuroscience concepts and an explanation for why this type of research is important for educational practice. We argue that basic principles of neuroscience and neuropsychology should be part of the knowledge base of teachers and integrated into teacher training. Teachers will benefit from knowledge and insights into the learning student because it may give them a new perspective to reflect on their pedagogical approach and professional experience and, thereby, their teaching.

## On Neuroeducation

### On the Science of Mind, Brain and Education and Related Fields

The focus in the present paper lies on the multidimensional domain of education and the possible relevance of insights from the science of *Mind, Brain and Education* (MBE, see Tokuhama-Espinosa, 2011). MBE is closely related to the new science of learning (OECD, 2007) and is also known as *Educational Neuroscience*. It is an interdisciplinary research field that seeks to integrate knowledge about the neural mechanisms of learning and development with insights from the field of education, and aims to improve our understanding of the way environmental factors influence brain structure and function, and thereby impact the conditions under which learning takes place. Accordingly, MBE is also a fundamental science that studies how education changes the brain (Ansari et al., 2017) and how interventions aimed at improving brain function can impact learning. It is therefore a misunderstanding that MBE research *primarily* aspires to improve educational practice and policy. Even so, the translation to education, which we refer to as *Neuroeducation* in the current article, is an important objective in the field of MBE. Generally, MBE, and neuroeducation in particular, aim to support the dialog between researchers and practitioners in the fields of neuroscience and education, and to encourage transdisciplinary partnerships (Sigman et al., 2014). Such partnerships have the potential to improve educational outcomes by integrating teachers’ practical experience with scientific insights into the mechanisms of attention, motivation, executive functions, and memory, and the effects of sleep, health, stress, and other conditions that influence learning.

It is important to note that the domain of neuroscience is vast, and extends far beyond the research approach in which brain structure and function are measured via brain imaging techniques such as Magnetic Resonance Imaging (MRI). Accordingly, it is better to speak of “the neurosciences”; the field encompasses at least 40 disciplines and subdisciplines, ranging from neurobiology, neuroanatomy, and neurophysiology to neurology, neuropsychology, and even neurophilosophy. Neuroscientists work together with many disciplines such as psychologists, health care professionals, philosophers, and educational professionals.

The science of MBE is closely related to and overlaps with *Cognitive Neuroscience*: the science that represents the convergence of cognitive psychology and neuroscience (Gazzaniga et al., 2008). Cognitive neuroscientists study the brain mechanisms underlying complex human behaviors such as language, learning, decision making and emotional processing. Likewise, MBE has links to the domains of *Affective Neuroscience* and *Social Neuroscience* (e.g., Immordino-Yang, 2011; see also Mills et al., 2014; Chen et al., 2018), which are connected to the field of Cognitive Neuroscience, but have their focus upon emotional processing and social behavior rather than cognition *per se*.

Another related field is *Developmental Cognitive Neuroscience*, which concentrates on the developing individual. Developmental cognitive neuroscientists study cognitive development and learning in relation to changes in brain structure and function (see Ansari et al., 2017; Thomas et al., 2019). For example, they evaluate how neural activation and task performance are influenced by developmental changes in cognitive functions such as attention (Posner and Rothbart, 2013), working memory, and executive functioning (Diamond, 2013). In addition, they investigate the influence of external, non-psychological factors such as sleep (Sharman et al., 2020) and exercise (Hillman et al., 2008; Mandolesi et al., 2018). Beside the study of cognitive functions, developmental cognitive neuroscientists also study the interaction with the social environment and the influence of emotional factors and motivation (e.g., Somerville and Casey, 2010; Mills et al., 2014; Blakemore, 2018). In fact, in the past few years there has been a tremendous increase in our understanding of “the social brain of the adolescent” (Crone and Dahl, 2012; Knoll et al., 2015), and its relation to natural curiosity, exploratory behavior and learning (Dahl et al., 2018; Steinberg, 2019).

Finally, the field of MBE is also related to the discipline of *Neuropsychology*. Neuropsychology is a behavioral science in its focus upon human behavior, cognition and emotion but also a neuroscience in that it strives to understand the individual by application of insights from neuroscience (Lezak et al., 2012; Kolb and Whishaw, 2015). Many applied neuropsychologists work with patients suffering from cognitive or behavioral problems in relation to brain dysfunction, developmental disorders, or cognitive aging.

The present paper places its focus upon Developmental Cognitive Neuroscience and Neuropsychology, but also on the basic neurosciences that are needed to understand the development and maturation of the brain and its impact on learning and educational achievement.

### The Challenge of Interacting Levels of Analysis

A major challenge that the field of MBE faces is that brain and behavior are studied on different levels of analysis (Willingham, 2009; Van Leijenhorst et al., 2017). At the lowest levels of analysis, neuronal processes and brain anatomy are examined in great detail, for example by recording of activity in individual neurons or by studying brain tissues and cells under a microscope. At higher levels of analysis, neuroscientists study brain structures or even entire brain networks, sometimes in relation to cognitive abilities. These cognitive abilities are generally studied in isolation, using well-controlled but relatively artificial tasks. Even higher levels of analysis focus on the child, the class, or the entire school system. Educational scientists find themselves on these higher levels of analyses, studying the child in interaction with its environment. On the higher levels of analysis, learning is often examined in naturalistic settings that unfold over the course of weeks or even months, rather than minutes or seconds, as is the general case in research on lower levels of analysis.

Because of the differences in granularity, complexity, and timescale, it is often difficult to draw inferences from one level of analysis to another (Willingham, 2009). Therefore, when translating findings from cognitive neuroscience to the field of education, it is important to realize that the “whole child” is more than the “sum of its parts.” Akin to a dish that gets a unique flavor due to the interaction between different ingredients, children cannot and should not be reduced to a collection of separate cognitive functions, affective tendencies, and personality traits, let alone a collection of neural predispositions. Moreover, just like the appreciation of food is influenced by the method of preparation, children are influenced by their immediate environment, including the family, their class, the teacher and school, as well as the broader society and culture that they grow up in. Contextual theories such as Bronfenbrenner’s bioecological systems theory argue that children participate in and are influenced by multiple interacting social contexts (Bronfenbrenner, 2005). Importantly, contextual influences can go all the way back to the level of the brain, and even to the level of genes (Gottlieb, 2007). Psychobiological research has revealed that there are bidirectional interactions between all levels of analysis, including genes, brain, behavior, and the social and cultural environment, suggesting that processes at lower levels of analysis should not be seen as causal factors driving functioning at higher levels of analysis, but as components of a larger dynamic system (Gottlieb, 2007; Greenberg, 2007). Therefore, when studying developmental changes, for example during the transition from childhood into adolescence, attention should be paid to all those multiple interacting levels of change, including physical, cognitive, and emotional changes, as well as changes in the social context and responsibilities (Dahl et al., 2018).

Educators have the difficult job of integrating all these different aspects of children’s functioning and behavior. Yet, in our opinion, this does not mean that knowledge of separate cognitive processes and neural mechanisms is not relevant to education. We argue that conceptual knowledge about the developing mind and brain could help teachers to look more systematically at their students, lessons, or classroom interactions. This would allow them to make more informed decisions about how to approach a particular situation. For example, research in the domain of cognitive neuroscience suggests that children benefit from an “enriched” learning environment, including activities that trigger curiosity and stimulate their language and thinking abilities, but neuroscientific findings also illustrate the importance of focused attention and preventing distraction (Dehaene, 2020). It is the teacher’s task to weigh these different insights, along with findings from other domains, and find the right approach for each child in each particular situation.

### Neuroeducation: Problems and Pitfalls

Through the years there have been criticisms on the notion that neuroscience knowledge could impact educational decisions and approaches. In his seminal paper, Bruer (1997) states that the translation of neuroscience to the field of education is “a bridge too far.” Likewise, in the past two decades, other authors have been skeptical about the relevance of neuroscientific findings for education, concluding that “only evidence from psychological experiments that examine behavior is relevant to education” (e.g., Bowers, 2016) and that “social problems require social solutions, not reduction to neural mechanisms” (e.g., Lalancette and Campbell, 2012; see Ansari et al., 2017 for discussion). Recently, Willingham (2018), discussed in Thomas et al. (2019) noted that knowledge of psychological theory or neuroscience findings is not necessary to teachers. According to Willingham, teachers should “understand children” and be able to observe the child in order to find patterns and consistencies in their cognition, motivation and emotion. On good grounds, however, others have argued that such a perspective is unnecessarily narrow (e.g., Howard-Jones, 2014; Horvath and Donoghue, 2016; Ansari et al., 2017; Thomas et al., 2019), given the multidimensional character of the educational domain. Nonetheless, it is important to emphasize that neuroeducation cannot and should not be *prescriptive*, in the sense that neuroscientific insights will be able to tell teachers what to do in a particular situation. Instead, neuroeducation should aim for *conceptual translation*, providing a broader context to understand the way children learn and develop. This could lead to better theories about education, and aid teachers in their decision making (e.g., Horvath and Donoghue, 2016). Or, as Schwartz et al. (2012) put it: “In education, there are few things as practical as a good theory,” referring to Kurt Lewin’s famous Maxim.

Besides arguments related to the relevance of neuroscience to education, there are also concerns about the reliability of the methodology and its practicality for describing the functioning of an individual subject (Thomas et al., 2019). As Thomas and colleagues describe, some of these criticisms are exaggerated by focusing solely on functional brain imaging, thereby negating the fact that the neurosciences involve a multitude of different domains, overlapping with behavioral and cognitive sciences. Recent papers from eminent researchers provide examples of neuroscientific evidence that are not derived from neuroimaging research but have major implications for education (see Fuhrmann et al., 2015; Diamond and Ling, 2016; Galvan, 2017; Goswami, 2019). Nevertheless, there are indeed limitations to neuroscientific methodology (which is also the case for other types of research), and it is of utmost importance that researchers remain cautious when interpreting and communicating their findings. Yet, awareness of methodological limitations should encourage rather than impede interdisciplinary collaboration. Only by integrating insights from different domains can we move the field forward.

### Neuroeducation: On Neuromyths, and the Seductive Allure of Neuroscience

A major problem in the application of neuroscience insights into education has to do with the so-called “neuromyths.” “Neuromyths are misconceptions about brain function generated by a misunderstanding, a misreading, or a misquoting of facts scientifically established (by brain research) to make a case for use of brain research in education and other contexts” (OECD, 2007; Dekker et al., 2012; Macdonald et al., 2017). As an example, the most persistent neuromyth (see Dekker et al., 2012) states that individuals should be taught according to their preferred learning style – whether they are a visual, auditory or kinesthetic learner. However, many research papers have been published in the past decade, showing again and again that learning styles do not exist (see Macdonald et al., 2017). A belief in this myth could be harmful to the learning individual as the “preferred learning style” does not always provide the best fit for the learning goal and hampers the development of experience with other learning strategies. This also applies to another often-mentioned myth concerning the idea that everyone is either a left- or right-brained learner (e.g., Dekker et al., 2012). So, the danger of neuromyths is that they are (inappropriately) applied to the classroom, leading to less effective teaching and learning. In addition to that, the discussion and uncertainty surrounding neuromyths could lead to a lack of confidence in the field of neuroscience and the many neuroscience facts which deepen our understanding of the learning process (this article, see also Dehaene, 2020). Therefore, educational professionals should become aware of the possibility that their convictions about neuroscience can have a negative impact upon their teaching. This implies that teachers should adopt a critical-reflective teaching method as suggested by Bergmann et al. (2017) and others.

Besides neuromyths, we also need to be mindful of the convincing power that neuroscientific findings may have on public opinion. Neuroscientists and educational professionals alike have warned us for the “seductive allure of neuroscience explanations” (e.g., McCabe and Castel, 2008; Weisberg et al., 2008) that may sway people’s opinion about political, legal, or educational issues or trap them into buying something that they do not need. Although others have shown that the seductive allure of neuroscience is not as ubiquitous as initially suggested (Farah and Hook, 2013), there are circumstances under which individuals are particularly prone to biased judgment when presented with neuroscientific evidence. For example, Scurich and Shniderman (2014) showed that people find neuroscientific evidence more persuasive when these findings are in line with their prior beliefs, suggesting that neuroscientific evidence may fall prey to the same thinking biases that are evident in the appraisal of other types of (scientific) evidence. Future research should further investigate the circumstances and individual characteristics that moderate the seductive allure of neuroscience, as this allure effect may contribute to the spread of neuromyths and biased judgment.

On the positive side, the fact that a belief in neuromyths is prevalent among teachers can be taken as an indication that they stand favorable to the notion that knowledge about the brain is relevant for learning and teaching. Interestingly, we have obtained strong indications that the prevalence of “believe in neuromyths” is highest in educational professionals who have the best knowledge about the brain (Dekker et al., 2012). We take this as an indication that it is difficult for teachers to find valid neuroscientific knowledge on the internet and in their professional literature. This underscores the notion that a valid and reliable knowledge base about neuroscience – neuroscience literacy – is urgently needed because having an understanding of neuroscience will enable educators not to use or promote misconceptions about the brain, and avoid the acceptance of educational products that cannot stand the critic.

### Example: The Appeal of “Brain-Training” Programs

It is probable that neuromyths and the seductive allure of neuroscience may have played a role in the popularity of so-called “brain-training” programs. These are computerized training programs targeting fundamental cognitive abilities, such as working memory and the executive functions (Diamond and Ling, 2016) which are described in section “Development and Training of the Executive Functions.” The idea of brain-training gained traction after initially promising findings of training-induced changes in cognitive task performance (see Jolles and Crone, 2012; Constantinidis and Klingberg, 2016), and even measures of fluid intelligence (Jaeggi et al., 2008). The impression that playing brain-training games could make you smarter and “unlock your brain’s full potential” was appealing to the general public. Hence, well before the scientific community had gathered enough evidence for its effectiveness, commercial parties started selling brain-training software. The claims that are made by these companies are often over-exaggerated and not backed-up by solid research (Simons et al., 2016). Moreover, the term “brain-training,” which is mostly used by commercial parties rather than by researchers themselves, is misleading as it suggests that neural changes are specific to this particular type of training. This completely pushes aside the fact that learning *always* changes the brain (Jolles and Crone, 2012). Besides, findings of training-induced modulation of brain function are largely irrelevant to the question of whether brain-training has any practical value beyond the lab. This point was also made by Simons and colleagues who published a comprehensive 82-page review article on the effectiveness of brain-training interventions. Their conclusion: brain-training frequently improves performance on the trained tasks and often on closely related tasks, but there is currently little evidence that it improves real-world outcomes (Simons et al., 2016). Yet, by the time this paper was published, the brain-training industry had become a multi-million-dollar business. It goes without saying that consumers are free to play brain-training games if they choose to do so, but the question is whether they would pass their time in a different way if they were sufficiently informed about the current scientific basis of these programs. This example illustrates the importance of careful communication of research findings to educators and the general public and investing in a greater (neuro)scientific literacy.

### Neuroeducation: Chances and Possibilities

Notwithstanding the critics mentioned above, there is a strong and positive attitude toward a new “science of learning” in which insights from the neurosciences, cognitive science, educational science and the behavioral sciences are merged (Sigman et al., 2014; Mayer, 2017; Thomas et al., 2019; Dehaene, 2020). Thus, scientific activities in the “Decade of the Brain” in the 1990s (Jones and Mendell, 1999) have led to major advances in the crosstalk between basic neurosciences such as neurobiology, neurophysiology, neurochemistry, neuroanatomy and others, and the exchange with applied disciplines such as neurology, psychiatry and clinical neuropsychology. This has yielded breakthroughs in our understanding of brain structure and brain function in normal conditions (e.g., in cognitive development and cognitive aging) and in pathology (e.g., many neurological diseases and psychiatric conditions). In addition, since then there has been a tremendous technological advance in the *in vivo* imaging of brain function, notably by functional MRI, and EEG techniques. This enabled researchers to investigate the human brain in action and has led to the theoretical advances that made the science of MBE viable as a field (e.g., Goswami, 2003; Ansari, 2005; Tokuhama-Espinosa, 2011). Brain imaging experiments informed us about mechanisms underlying the processes of reading, arithmetic and other academic achievements (see Dehaene, 2020) and provided clues as to the neuroscientific basis of conditions such as dyslexia, dyscalculia, ADHD, depressed mood and anxiety-related problems. Yet, up till today, the findings from cognitive neuroscience are considered to be important for progress on the scientific domain but not yet sufficient to be of direct help in the design of innovative teaching techniques and didactics and in educational interventions (Ansari et al., 2017). The promise of neuroscience research for the field of education may lie more in the use of insights related to the interaction between learning and development and in the internal and contextual factors that impact learning, including the application of pedagogical principles (Thomas et al., 2019; see also Tokuhama-Espinosa, 2014). Many of these ideas are grounded in a broader evolutionary framework which suggests that learning and development are evolved features allowing the individual to adapt to their current and future environment (Bjorklund, 2018, 2020). The key insights are outlined in section “BRAIN DEVELOPMENT, LEARNING AND THE NOTION OF PLASTICITY” below in which we focus on principles of learning and development and domain-general skills. The following chapter (see section “WHAT EDUCATIONAL PROFESSIONALS NEED TO KNOW ABOUT NEUROSCIENCE”) formulates a proposal as to the nature of the neuroscientific knowledge and insights which could be of use for educational professionals, and elaborates on the possible approach that is to be adopted.

## Brain Development, Learning and the Notion of Plasticity

### The Brain as the Engine for Learning

Evolution has equipped the human brain with a number of important learning mechanisms that allow the individual to efficiently take in new information and adapt to the ever-changing environment (see Kolb and Whishaw, 2015; Kalat, 2018; Dehaene, 2020). For one, the brain is responsible for children’s natural curiosity and exploratory behavior, which drives the development and organization of cognition and behavior in relation to environmental demands (Jolles, 2016, 2020). Furthermore, attentional mechanisms guide the orientation to stimuli in the physical, cognitive and the socio-emotional domain. They are responsible for amplifying important signals while discarding stimuli that are not relevant for current or future use (Dehaene, 2020). The selection that the brain makes is based upon past experience and evaluation of the possible future use. As such, the brain constantly makes predictions about what is going to happen and what would be the best way to act. Errors in such predictions are used to update mental models of the environment. Finally, the brain selects the stimuli that will be consolidated into memory for use at a later moment in time (see also Dehaene, 2020).

Information processing and the processes of attention and consolidation are quite well understood, and the neurochemical, neurobiological, and neurophysiological principles underlying it are currently handbook knowledge for students in the neurosciences and biological psychology (e.g., Kolb and Whishaw, 2015; Kalat, 2018). The same applies to the process of retrieval of stored information from memory. A broad understanding of the brain’s attention, consolidation and retrieval processes is not only relevant for the remediation of patients with a neuropsychological dysfunction or brain disease (see Lezak et al., 2012) but also for application on children and adolescents in their development and schooling. Yet, it is important to take note of the biological and contextual factors that constrain these processes.

As the brain is part of our body and an organ in need of energy, nutrition and sensory stimulation, it is subject to metabolic constraints. Energy, nutrition and sensory information are therefore needed to ensure that the brain is in an optimal condition to learn (see also Thomas et al., 2019). So, when the brain functions sub optimally, it can experience problems in learning and attention which could manifest themselves in forgetting, lack of concentration, academic indifference or cognitive overload. Many contextual and internal factors have been found to impact optimal or suboptimal functioning of the brain (e.g., Lederbogen et al., 2011; Batenburg-Eddes and Jolles, 2013; Goddings et al., 2014; Miller and Halpern, 2014; Smith, 2018; Sharman et al., 2020) including: (lack of) sleep, fatigue, problems in energy supply, metabolic problems, puberty, sex differences, dietary intake, stress and/or major affective problems (mood problems, aggression, anxiety), use of alcohol and drugs, sensory under- or overstimulation, and developmental dysfunctions. Therefore, and because of their influence on brain functioning, these external and largely non-psychological factors can have a major impact on educational outcomes. This is the reason that researchers on the domain of MBE in past years have investigated educational interventions and learning performance in relation to contextual and supportive factors such as sleep (Sharman et al., 2020), the effect of nutritional interventions (Wurff et al., 2019), the impact of movement and physical exercise (Heppe et al., 2016; Reigal et al., 2020), mindfulness training (see Felver et al., 2016), action video game playing (Bediou et al., 2018), learning a musical instrument or a second language (Moreno et al., 2015; Benz et al., 2016), and others.

The notion behind educational interventions such as mentioned above is that the student should arrive in the learning situation *fit to learn*: be it listening to instruction in the classroom, working on homework assignments or exploring a museum. In other words, the brain should be in an optimal condition for information processing (see also Thomas et al., 2019). Interventions such as those mentioned here are thought to help in attaining this goal by stimulating active engagement, optimizing information processing, focusing attention and sustaining concentration. This enables the student to get more study motivation and improve in academic performance. According to leaders in the field, this type of neuroscience findings are potentially able to enrich theories of cognition and behavior. It is promising in this respect that new resources pertaining to neuroscience findings have become available to teachers in past years: online courses and books on topics related to brain function and development, and on behavior both in- and outside the classroom (e.g., Tokuhama-Espinosa, 2011; Steinberg, 2014, 2019; Tokuhama-Espinosa, 2014; Blakemore, 2018; Hohnen et al., 2019).

### The Development of the Brain

The development of the central nervous system starts already very early in the prenatal period, but major changes still take place in the micro- and macro architecture *after* birth. Both prenatal and postnatal development are subject to environmental influences. Of major importance in the postnatal period is the finetuning in the development of the *neuronal networks*, which connect the many regions in the brain cortex and structures deeper in the brain. This period is characterized especially by the major changes in connections between adjacent cells and the fine-tuning of connections within and between neuronal circuits.

The networks enable the brain to act like a symphony orchestra whereby individual regions in the brain can contribute to the total output by working together with other regions that have another task or role. In particular developmental periods – notably in early childhood and early adolescence – there is a burst in the number of synapses and connections that neurons make, followed by a period of elimination (called “*pruning”*) of some of these connections and synapses. Synaptic plasticity (see section “Brain Plasticity Is the Key to Learning and Development”) is thought to be one of the primary mechanisms by which the brain changes as a function of experience and which results in learning. Another mechanism is that of *myelin formation* in which particular bundles of axons become insulated by a specialized non-neuronal cell, and this results in faster electrical transmission over these fibers.

The maturation of the brain is thought to proceed up till well after the 20th year of life (Gogtay et al., 2004; Giedd, 2015; Dahl et al., 2018; Steinberg, 2019). The initial overgeneration of synapses and their elimination (synaptogenesis and synaptic pruning) are not uniform across the brain but they differ by regions. It appears that regions associated with basic motor and sensory functions undergo these developmental processes earlier in the period of childhood and adolescence than regions involved in higher-level functioning (to be described in par 3.6. in terms of Executive Functioning). Therefore, regions whose functions are heavily affected by experiences and new knowledge – and thus by learning and education – are relatively late to mature. This applies to regions within the prefrontal and parietal cortices and the neuronal networks which they are part of (e.g., Crone and Dahl, 2012; Blakemore, 2018). So, the various regions in the brain (and their connections) mature according to a different time scale.

### Brain Plasticity Is the Key to Learning and Development

The brains of different people generally have the same large-scale organization which has evolved over the course of evolution. Yet, although the basic structure and functioning of the brain is influenced by genetic predispositions, there is a built-in flexibility in brain development. This allows the brain to adapt to its specific surroundings, thereby enhancing the chances of survival and optimal behavioral adjustment (Bjorklund, 2020; Dehaene, 2020). This built-in flexibility is called “plasticity,” a key neurobiological process that refers to neural changes in response to experience and to specific characteristics of the (internal and external) environment. Plasticity allows the brain to reorganize after injury and to adjust to atypical environmental circumstances. Yet, plasticity is also key to typical development and learning at home and in school. The fact that the brain is able to change in response to environmental demands makes learning and education possible. This is essential for the individual to adapt to a changing environment.

It is relevant to distinguish between the so-called “experience-expectant plasticity” and “experience-dependent plasticity,” as proposed by Greenough and colleagues in 1987. *Experience-expectant plasticity* refers to neural changes in response to experiences that are universal to all individuals within a species, such as changes related to the perception of light and sound. At birth, brain regions have a certain predisposition for specialized processing in a specific domain (e.g., visual or auditory perception, spatial processing, or language) by virtue of the cell types and connectivity patterns that these brain regions display (Dehaene, 2020). Yet, specialization will only occur if the individual receives the right kind of stimulation within a certain time frame of development. If the right input is not received, this will result in an abnormal pattern of neural organization (Greenough et al., 1987). At first sight, this may seem inefficient and even potentially harmful. Yet, this type of plasticity allows for greater flexibility in unusual circumstances, e.g., in case of sensory impairments such as blindness, while providing enough stability once the brain matures (e.g., Bedny et al., 2015).

In contrast, *experience-dependent plasticity* occurs in response to experiences that vary between members of a species, i.e., the culture in which the subject grows up or the skills they acquire in school or on the sports field. Examples are academic skills, such as learning how to read and do math, learning to appreciate literature, interests and activities in the domain of science and technology, playing football or studying the history of the country. Experience-dependent plasticity thus allows individuals to adapt to their unique environments by impacting the biological organization of the brain of the individual. Importantly, experience-dependent plasticity is not strictly age-dependent, allowing changes across the life span. Nevertheless, it is important to keep in mind that plasticity is generally greater early in life, and that early environmental influences may influence future developmental trajectories (Bjorklund, 2020). This explains how slight differences between individuals in their preference, information processing abilities, or contextual input at an early age may cascade into larger differences later on.

The examples show: *it is the context that shapes the brain* (Jolles, 2016). Whereas genetic predispositions are responsible for innate perceptual, cognitive or behavioral biases, the particular experiences and socio-cultural niche in which learners find themselves determine the way in which such biases are expressed and develop into more complex psychological mechanisms (Bjorklund, 2020). As such, the concept of Gene X Environment X Development interactions (cf. Bjorklund, 2020) is the essence of the learning process and of key importance to teachers, as they have the task to provide the optimal conditions for learning, and decide about the timing of instruction (Thomas and Knowland, 2009). Teaching and the pedagogical approach chosen by the teacher will enable their students to encode knowledge, make creative connections between different pieces of information, to acquire basic academic skills and to broaden their knowledge about the world.

### Examples of How Experience Shapes the Brain

The brain reacts to environmental stimulation by an adaptation of its macro- and microarchitecture. In past decades, research evidence obtained in animals but also in human subjects has shown that the organization of complex neuronal networks in the brain can change in relation to sensory stimulation, execution of simple and complex motor acts, and other types of experience (e.g., Jolles and Crone, 2012; Ansari et al., 2017; Dehaene, 2020). As an example mentioned by Ansari et al. (2017): when one of the fingers is consistently stimulated more than the others, its representation in specialized structures on the brain cortex will eventually be enlarged relative to the cortical representation of the other fingers. Eventually, more neurons in a specialized region in the motor cortex will respond to the stimulated finger in comparison to the non-stimulated ones. Similar findings have been shown in the domain of music learning in string instrument players (e.g., Pantev et al., 2003). Other well-known examples are changes in brain structure in subjects who learn to juggle (Draganski et al., 2004) and in taxi drivers who learn to navigate complex spatial environments (Maguire et al., 2006). Furthermore, it has been shown how the brain changes in relation to learning to read, learning to do arithmetic, and learning other types of auditory, visual and language skills (see Dehaene, 2020 for elaboration and Ansari et al., 2017). Importantly, learning-related changes have not only been observed *within* individual brain regions, but also in the interactions *between* brain regions (e.g. Mackey et al., 2012, 2013; Jolles et al., 2016; see also Jolles et al., 2020). This is in line with the idea that functional specialization of brain circuits occurs through activity-dependent interaction and competition between different brain regions (Johnson, 2011). In this context, it is important to reiterate that experience-related changes in brain function and structure should be viewed form a developmental perspective, suggesting that experience-dependent plasticity is not necessarily the same for children, adolescents and adults (for a more extensive discussion of maturational changes in learning and plasticity, see Galvan, 2010; Johnson, 2011; Jolles and Crone, 2012). Finally, besides direct effects on neural processing within and between specific brain areas, experience may also have more general or indirect effects on brain function. Of particular interest in this respect is the effect of physical exercise, which appears to benefit cognitive functioning and wellbeing by inducing more broad neurobiological changes (for a review, see Mandolesi et al., 2018).

### Psychological and Social Factors and the Brain

In recent years, new scientific knowledge has been obtained which shows that individual differences in children’s socio-economic status (SES) and the environment in which a child grows up affect the organization of the human brain (Hackman et al., 2010; Ansari, 2012; Farah, 2018; see also Rindermann and Baumeister, 2015). As an example, Lederbogen et al. (2011) showed that the brain’s response to stress was different in individuals growing up in urban environments versus those growing up in rural environments. There is now strong evidence that the brains of children growing up in environments that do not supply the proper sensory, cognitive or social-emotional stimulation develop differently from those of their peers who grow up in more “enriched” environments. Several papers have been published which make this point in a comparison of children growing up in families from a lower versus higher SES (e.g., Mackes et al., 2020; see also Farah, 2018). As an example, Khundrakpam et al. (2019) found non-linear effects of socioeconomic status on brain development in childhood and adolescence with associations between parental occupation, cortical thickness and language skills. In adolescence, social isolation appeared to disrupt cortical development and goal-dependent decision making (Hinton et al., 2019). Likewise, both brain structural and functional changes were apparent in adolescents in the context of alcohol abuse (Jadhav and Boutrel, 2019). It has been concluded that brain maturation is negatively affected by poverty (see Noble, 2017; Davis, 2020). The findings from a rapidly increasing number of research articles thereby underscore the vision that the social environment is very important, and – in relation to the many findings about brain plasticity (see section “Brain Plasticity Is the Key to Learning and Development“) – the notion that *context shapes the brain*. It is of interest that similar findings had already been done in the fifties and sixties in rodents. These investigations showed that rats that were reared in impoverished environments had smaller brains than rats who were reared under enriched conditions (see textbooks such as Kalat, 2018 and Gray and Bjorklund, 2018).

In the context of education, it is of importance to note the large literature on possible sex differences in cognitive performance and the question as to whether differences are due to biological factors or to culture and the social environment. Strong indications exist that both biological and social/cultural factors play their role. This implies that the differences in cognitive performance which have been reported in the many scientific articles which are based upon crosssectional research can*not* be ascribed to inborn mechanisms *per se*. Accordingly, Miller and Halpern (2014) in their authoritative review on “the new science of cognitive sex differences” elaborated upon the important role of upbringing and cultural factors such as economic prosperity and gender equity, but also on brain factors and the role of prenatal androgens. With respect to differences in brain structure, Lenroot and Giedd (2010) showed that adolescent males and females exhibit a four years difference in the age at which their brains reach the greatest volume (the average age is 10.5 years for females and 14.5 years for males). Thich implies that the brain maturation of males lags behind that of females in the period of early and middle adolescence (see also Gur and Gur, 2016). The notion that brain maturation of boys and girls follows another timescale receives support from other investigations (see Miller and Halpern, 2014; Giedd, 2015; Choleris et al., 2018; van der Graaff et al., 2018; van Tetering et al., 2018; Wierenga et al., 2018). Such a maturational gap is thought to make the brain development of boys and girls differentially vulnerable to upbringing and the influence of their environment – which is different for the majority of boys and girls from birth on (Miller and Halpern, 2014; Jolles, 2016, Jolles, 2020). This explains findings such as those reported by Barbu et al. (2015). These authors studied sex differences in language acquisition across early childhood and found that family socioeconomic status does not impact boys and girls equally. Likewise, the sex differences in self-regulation in adolescents which we recently found in a large-scale cross-sectional study could be ascribed to the major influence which social factors have on brain maturation (van Tetering et al., 2020). Taken together, boy-girl differences in cognitive performance and academic achievement are due to a complex interplay between biological and psychosocial factors. It is thus of importance to understand how biological and environmental factors interact and, as Miller and Halpern (2014) put it “in order to maximize cognitive potential and address pressing societal issues.”

The findings are of major importance for the domain of education. This is because of the challenges that teachers encounter in their educational interactions with boys and girls, with students who have another cultural background, and those who differ in socio-economic factors and the financial possibilities of their parents (e.g., Rindermann and Baumeister, 2015). More research is needed, but the studies which have been performed up till now do suggest that personal life- and learning experiences and culture are an important factor that impacts neuropsychological functioning. While education plays an important role in passing on cultural norms and values, there are also cultural differences in the way education is organized (see Downey et al., 2019). Cross-cultural research suggests that this may influence the development of cognitive and academic skills, including executive functioning (e.g., Ellefson et al., 2017; Xu et al., 2020). Taken together, it is probable that these socio-cultural factors impact the extent to which the developing child has been stimulated on the physical, the cognitive, the social and the emotional domain (Jolles, 2016), leading to differences in brain function across children from a different background. The extent to which specific cultural and economic factors impact brain development is an important direction of future research.

### Childhood, Adolescence, and Emerging Adulthood

Throughout the past decades there has been a significant amount of scientific investigation into brain development across childhood and adolescence (see Crone and Dahl, 2012; Sheridan and McLaughlin, 2014; Fuhrmann et al., 2015; Blakemore, 2018; Dahl et al., 2018). As described in Box 1, research shows that various brain regions display a different developmental trajectory, with regions in the temporal and frontal lobes the last to mature. Knowledge of these regional trajectories offers insights into the developmental timing of emerging skills related to decision-making, perspective taking, self-regulation, and other major cognitive and affective functions (e.g., Crone and Dahl, 2012; Mills et al., 2014; van Tetering and Jolles, 2017; van Tetering et al., 2020; see also the paragraphs on Executive Functioning later in this chapter). A number of important changes take place during adolescence, a distinct developmental period characterized by rapid growth, hormonal and metabolic changes, specific neuro-maturational changes, as well as changes in social and cultural responsibilities (Crone and Dahl, 2012; Blakemore and Mills, 2014; Dahl et al., 2018; Steinberg, 2019). Growing evidence points to a particular importance of changes in social and affective processing during adolescence (e.g., Larsen and Luna, 2018). Importantly, insights about changes in sensitivity to the peer group and social rewards are crucial for understanding adolescent vulnerabilities such as the high rates of risk-taking and substance use (Knoll et al., 2015; Romer et al., 2017; Smith, 2018). Yet, adolescence is also a window of opportunity for social and emotional learning, and making a positive impact on societal problems (UNICEF, 2017; Dahl et al., 2018). It is becoming acknowledged that the adolescent brain is a social brain (Crone and Dahl, 2012; Blakemore and Mills, 2014; Blakemore, 2018), which is open to novelty and exploration, and thus for knowledge acquisition and learning new skills (Batenburg-Eddes and Jolles, 2013; Fuhrmann et al., 2015; Jolles, 2016). This makes learning an important target for interventions not only on the domain of cognitive performance but also – and especially – that of social and emotional learning (Blakemore, 2018). Therefore, the evidence pointing to the prolonged brain and neuropsychological development across adolescence has a profound influence on the way in which we now think about the teens who traverse this important phase.

**Box 1. Basic building blocks of the brain.**

To better understand and appreciate the insights summarized in section “BRAIN DEVELOPMENT, LEARNING AND THE NOTION OF PLASTICITY,” it is necessary to have a basic understanding of the “basic building blocks” of the brain and their development. For this, we would like to refer to a report by the Society for Neuroscience (SfN), formulating eight “*Neuroscience Core Concepts (The Essential Principles of Neuroscience)*” that one should know about the brain and nervous system, and have broad application for K-12 teachers and the general public (Society for Neuroscience, 2008; Note: K-12 means ‘from Kindergarten to 12th grade and is an American expression which indicates the range of years of publicly supported primary and secondary education found in de United States). This text summarizes what every student – and of course their teacher – should know about neuroanatomy and the basic building units of the brain, and about its development and maturation. Briefly, everybody should know about the architecture of the brain and its basic constituents, the more than 90 billion neurons. *Neurons* are the nerve cells which underly brain function and eventually the biological functioning of the body, behavior, cognition and affect. Neurons have a cell body and several extensions, one of which is called the *axon*, through which the neuron sends electrical signals away from the cell body and others, called the *dendrites*, through which the neuron receives information from other neurons. The neurons communicate with each other via their axons and their connections on the dendrites and the cell body of other neurons. In the course of brain maturation, many nerve fibers eventually form highly interconnected *networks*. The points where nerve cells connect is called the *synapse*, and the *communication* between neurons takes place by biochemicals called *neurotransmitters*. The number of synaptic connections originating from one particular neuron can change in relation to experience and also the efficiency of the synaptic transmission can change. Being engaged in a complex neuronal network may lead to a situation in which the individual neuron eventually can have far more than 10.000 connections to other neurons.

The period of adolescence is thought to last from around 10 years of age to the mid-twenties (e.g., Steinberg, 2014, 2019; see also Crone and Dahl, 2012). While the beginning of adolescence is clearly marked by the onset of puberty, the end of adolescence is less clear (Giedd, 2015; Dahl et al., 2018). Late adolescence overlaps with adulthood in the phase of “emerging adulthood.” As proposed by Jeffrey Arnett (2000), this is an important period of development, in which the brain is still in a process of maturation, albeit less pronounced than before. Studies in which brain structure was measured by MRI, reveal that the brain continues to change in structure through emerging adulthood (Gogtay et al., 2004; Houston et al., 2014; Giedd, 2015; Galvan, 2017). Furthermore, emerging adulthood is a phase during which individuals gain important experiences related to the formation of their identity and “personal growth” (Hochberg and Konner, 2020). Therefore, it is now established that the human brain is not fully developed by the time individuals reach culturally defined adulthood – at the 18th birthday in many western countries. Experts in the field propose that individuals in their late adolescence and early adulthood sometimes do not yet have the skills, the attitudes and experience they need to act as an independent and well-functioning member of the adult society (Hochberg and Konner, 2020).

### Executive Functioning

In past years, we have gained much insight in a particular set of neuropsychological skills that function across cognitive domains and which are considered an essential prerequisite for learning and our adaptation to a changing environment. These so-called “Executive Functions” (EF’s) are a set of cognitive and non-cognitive processes that determine which sensory stimuli are selected and how information is processed, encoded and retrieved. They are essential for learning and have – over the past decade - received much interest from the educational domain (Jolles, 2016, 2020). Three important fundamental processes which are nowadays shared under the umbrella of the EF’s are working memory, inhibitory control and cognitive flexibility (Diamond, 2013). *Working memory* refers to the ability to hold information in a temporary storage while operating on it, whereas *inhibitory control* is the ability to inhibit responses and select among different stimuli that are present. *Cognitive flexibility* refers to the ability to switch back and forth between different tasks or perspectives (Diamond, 2013). The construct of EF shows overlap with different aspects of attention, including *focused attention*, which describes the ability to focus upon a particular stimulus while ignoring or inhibiting other types of information, and sustained attention, which refers to the skill of staying in a state of concentration for a prolonged period of time. Together, these basic neurocognitive functions enable the individual to engage with the material which has to be learned, to hold it in mind, operate upon it, and select the relevant information while at the same time inhibiting information that will not inform their understanding, but rather interfere with it.

The three fundamental EFs are controlled by higher order cognitive and non-cognitive functions or skills. As Adele Diamond (2013), an expert on the field of developmental cognitive neuroscience put it: “*Executive functions refer to a family of top-down mental processes needed when you have to concentrate and pay attention, when going on automatic or relying on instinct or intuition would be ill-advised, insufficient, or impossible.”* The EFs give us the time to think and not to act too quickly and enable us “to play with ideas.” They help us to engage in new, unexpected challenges, to resist temptations and to monitor the route to the goals we have formulated (Jolles, 2016; van Tetering and Jolles, 2017; van Tetering et al., 2020). When considering EFs in the broadest sense of the word, other relevant skills that fall under the umbrella of the EFs include self-insight and self-regulation, social monitoring, emotional processing and empathy, planning and prioritizing, overseeing the consequences of one’s actions and insights into other person’s intentions and the roles played in social and cultural contexts (Lezak et al., 2012; McCloskey and Perkins, 2013; Dekker et al., 2016; Jolles, 2016, 2020; Chen et al., 2018). The EFs play a key role in the evaluation of the emotional and motivational value of stimuli and they enable the individual to make plans for the short and long term, to prioritize and select the optimal route to attain goals and to be creative (Lilly, 2020). They enable the individual to evaluate or judge his or her position in relation to others, to the group and the social system and to act according to this evaluation. Therefore, the EFs are not only relevant for cognitive performance but also for self-regulation and behavior, and for social and emotional functioning. They are indispensable for personal growth over the period of childhood and adolescence.

Therefore, it goes without saying that the EFs are important for education. The EFs may help teachers to better understand students in their classroom, their behaviors and individual differences therein (e.g., Dawson and Guare, 2018). For effective teaching, teachers must be aware of how to get their students’ attention, how to inspire them and how to support the self-insight and self-regulation which are needed for study motivation and academic achievement. Teachers should have the tools and experience to alert their students, help them select the most relevant information, resist distraction, and encourage them to keep on task (i.e., to sustain their attention). Furthermore, teachers should have the know-how to help their students organize and prioritize in task execution and planning, and to support personal growth. Educators should therefore be aware of the existence of the EF and the role they play in learning and performance.

### Development and Training of the Executive Functions

Brain networks underlying EFs involve various substructures in de prefrontal cortex, in the parietal lobe, the limbic system and various subcortical regions which change over the course of an individual’s development (Morton, 2010; Larsen and Luna, 2018). The most fundamental EFs including the attentional processes and the ability to hold a select number of items in working memory start to develop already in very young children. The more complex processes, such as inhibitory control and the manipulation of information in working memory, develop over the whole period of childhood and adolescence (Hoeschler et al., 2018). Furthermore, higher-order cognitive and non-cognitive EF mature even through emergent adulthood in the third decade of life (e.g., Dahl et al., 2018; Steinberg, 2019). In the context of adolescence as the period in which the social brain develops it is not surprising that the non-cognitive aspects of EF become particularly important during this phase of life. The prolonged development of EFs makes these functions an important target for educational interventions (Thomas and Knowland, 2009; van Tetering and Jolles, 2017; van Tetering et al., 2020). Moreover, the finding that EFs differ between children depending on their cultural environment (e.g., Ellefson et al., 2017; Xu et al., 2020) suggests that these skills are changeable and plastic, and potentially trainable (Diamond and Ling, 2016, 2020; García-Madruga et al., 2016; Rice, 2016). This is also apparent from intervention studies showing that EFs can be remedied in children raised in deprived home environments (Neville et al., 2013). As the EFs are domain-general skills important to virtually all academic domains, it has been argued that targeting the EFs may have broad effects on academic development (Thomas and Knowland, 2009). Yet, more research is needed to find the most optimal ways to train EFs and potential moderating factors. At present, most training programs focusing on basic EFs show only limited transfer beyond the skills that are trained (Simons et al., 2016; Gathercole et al., 2019). Programs targeting higher-order EFs, notably self-regulation may have more potential in that respect (Poon, 2018; Xue et al., 2018: see also van Tetering and Jolles, 2017; van Tetering et al., 2020).

## What Educational Professionals Need to Know About Neuroscience

### Where Do We Stand?

The OECD stated its influential report “*Understanding the brain. The birth of a learning science”* (2007) that the time is ripe to use knowledge and insights about brain, cognition and behavior on the educational domain. Nowadays, fifteen years later, the field of MBE is still considered to be “promising” in its possible contribution to educational innovations. However, there is not yet a converging view on the nature of the knowledge and insights into brain and brain functioning which might have relevance for education. Accordingly, it is not clear what the best approach could be to educate the educator in this respect. An important reason may be that present insights into the basic architecture and mechanisms of brain and mind are huge and diverse. This makes transfer difficult: the knowledge is distributed over more than 40 neurodisciplines and over the fields of cognitive science, psychology and pedagogy. In addition, there has not yet been enough interaction between educators and educational scientists on the one hand and the various representatives of the neurosciences, the cognitive and behavioral sciences on the other.

As described in this article and in other papers on “the promise of the neurosciences for education,” the results from brain imaging research are considered to be very interesting and to have potential to contribute to our understanding of the mechanisms underlying activities such as language acquisition, reading, arithmetic and many cognitive functions and processes. However, the brain imaging findings as such are, generally, not able to provide the insights and predictions that the field of education needs on a day-to-day basis. There are very few examples of insights from brain imaging research that will directly contribute to innovations in didactics or teaching or provide guidance for the type of decisions that the teacher has to make in class. Yet, there is other neuroscientific knowledge that could be relevant for application in the educational domain: neuroscientific knowledge and insights could support teachers in their pedagogical approach by broadening our insights into the mechanisms of learning and the learning individual. The insights in brain plasticity and the factors which impact the optimal functioning of the brain may help to formulate answers to important questions like “what are the factors that determine the selection, consolidation and retrieval of environmental stimuli?”, “how does the brain learn from errors, and what is the role of surprise?”, “what external factors determine the efficiency of information processing?”, and “what are the optimal conditions for learning?”, as well as “how does the brain develop and mature over the long period from early childhood through emergent adulthood?”, and “how do educators (teachers, parents) influence that process?”. These issues about the brain are relevant for every educator.

### On Issues About the Brain That Every Educator Should Know

Section “BRAIN DEVELOPMENT, LEARNING AND THE NOTION OF PLASTICITY” of this article gives an introduction into the important theme of “plasticity” which is an inherent property of the brain that enables us to adapt to the ever-changing environment, and about the basic building blocks of the brain which underly plasticity (Box 1). “Experience-dependent brain plasticity” was described as the key process when the learning individual consumes new information and consolidates this into the brain hardware (i.e., in the extremely extended system of synaptic connections that make up large-scale brain networks). Complex information from the sensory, cognitive, social and emotional domain – i.e., the environment – interacts with genetically defined predispositions, and together they are responsible for brain development and learning. This underscores the notion that teachers, parents and other educators are important, even essential, for personal development. Educators create the conditions for the acquisition of knowledge and experiences that are to be stored by the learning brain and they inspire and direct the process of curiosity and information processing by the student (Jolles, 2016, 2020).

Specific attention should be paid to the executive functions. The most fundamental components of executive functions, including certain attentional and inhibition processes, start to develop already in early childhood. Yet, more complex EF abilities, notably self-insight and self-regulation, but also empathy, social monitoring, mental manipulation, cognitive flexibility, planning and problem solving, develop over the long period of childhood and adolescence – provided that the environment gives the support and inspiration that the learning person needs. In this respect, it is of major importance for the educational field to know that brain maturation extends through early, middle and late adulthood and toward the 23rd to 25th year of life. The major EF still develop, even in emerging adulthood. This reflects itself in the personal growth of the learning person, which enables him or her to take an independent position in society.

### Four Key Issues About the Brain

Based upon the accumulating insights described in preceding paragraphs, we will now describe four issues that – in our opinion – are to be regarded as essential knowledge for educators and which should be part of any teaching program for aspiring teachers and for continuous education. It is our conviction that knowledge of these issues will impact teaching and the pedagogical approach to the learning individual, and their development and personal growth. These four major issues around brain structure and neuropsychological functioning follow the eight “Neuroscience Core Concepts” (“The essential principles of Neuroscience”), which have been formulated by the Society for Neuroscience (Society for Neuroscience, 2008) and introduced in Box 1. These Core Concepts “offer fundamental principles that one should know about the brain and nervous system” (Society for Neuroscience, 2008). According to the SfN, the Neuroscience Core Concepts “have broad application for K-12 teachers and the general public, offering the most important insights gained through decades of brain research”.

The core concepts have to do with the four overarching insights that: (1) “The nervous system controls and responds to body functions and directs behavior”; (2) “Nervous system structure and function are determined by both genes and environment throughout life”; (3) “The brain is the foundation of the mind”, which includes cognitive, social and affective functioning; (4) “Research leads to understanding that is essential for development of interventions for the active stimulation of optimal brain function and therapies for nervous system dysfunction.” Note that the last statement was slightly adapted by the current authors to include the pursuit for optimal brain functioning in healthy individuals. Embedded in the four mega-concepts, are insights such as “the human brain endows us with a natural curiosity to understand how the world works” and “intelligence arises as the brain reasons, plans, and solves problems,” “life experiences change the nervous system” and quite some others which lie at the core of the issues described in the present article. We propose that the neuroscience core concepts formulated by the SfN could be a valuable starting point for any undertaking directed at “educating the educator” about the student and the learning brain. We consider this essential knowledge to be taught to both pre-service teachers and in-service teachers and other professionals in the educational domain. Box 2 goes in depth as to knowledge and insights that should be part of any undertaking at educating the educator about the brain (note that Box 1 confines itself to the description of the basic building blocks of the brain, i.e., neuroanatomical issues).

***Theme 1. “The nervous system controls and responds to body functions and directs behavior.”***

*This theme includes basic knowledge about the anatomy and functions of the nervous system (see also Box 1). Key topics include*:

**Box 2. Themes about brain functioning which should be part of the knowledge base of educators.**

It is of importance for the educational professional to have a basic insight into brain plasticity and brain development and into major aspects of human information processing. Textbooks for undergraduate students in psychology/behavioral science (e.g., “Introduction into Biological Psychology” Kolb and Whishaw, 2015; Gray and Bjorklund, 2018; and Kalat, 2018) may provide a good starting point. The four themes described below give a compact description of the issues which could be relevant in this respect. These topics are based on the “Neuroscience Core Concepts,” formulated by the Society for Neuroscience (Society for Neuroscience, 2008).

The micro-anatomy of the nervous system: cells, dendrites, axons, spines, glial cells, myelin, neurotransmitters, neurohormones. The macro-anatomy of the nervous system: hemispheres, neocortex, gray and white matter, cerebellum, basal ganglia, limbic system, thalamus and hypothalamus, brainstem and ascending/descending fiber system, blood supply of the brain. Neurophysiology, impulse propagation, synaptic transmission. The input and output systems: senses and incoming information; the peripheral nervous system, innervation of the muscles, endocrine glands and internal organs.

***Theme 2. “Nervous system structure and function are determined by genes and environment throughout life.”***

*This theme concerns issues related to brain development and the influence of experience. Key topics include:*

Brain plasticity. Brain development and maturation. Sensory circuits bring information to the nervous system whereas motor circuits send information to muscles and glands. Synaptic pruning. Development of child and adolescent through emergent adulthood. Sexual development. Individual differences. Organization of information processing, selection of stimuli, consolidation and retrieval. Natural curiosity and adaptation to a changing environment. Experiences change the brain. Lifelong changes in neuronal circuitry in relation to acquired knowledge and experiences.

***Theme 3. “The brain is the foundation of the mind.”***

*This theme concerns knowledge from the fields of cognitive (neuro)science and neuropsychology. Key topics include:*

Basic functions of the brain: motor function, impulse control and cognitive flexibility, sensory systems, perception, attentional functions and concentration, memory, learning and forgetting, language. Executive functioning: self-insight, self-regulation, social monitoring, emotional processing and empathy, anticipation of future actions, planning, prioritizing and problem solving. Higher functions and neuropsychological processes: intelligence, reasoning and thinking, identity formation, communication, motivational processes, curiosity, and imagination.

***Theme 4: “Research leads to understanding that is essential for development of therapies for nervous system dysfunction and helps improve the circumstances under which people learn.”***

*This theme involves a basic understanding of neuroscientific research methodology and scientific discovery. Key topics include:*

A basic understanding of the different disciplines within neuroscience, as well as other fields that intersect with neuroscience. The levels of analysis. Basic knowledge about methods used in brain and neuropsychological research in humans, notably Magnetic Resonance Imaging, EEG techniques, controlled experiments and quasi-experimental designs, epidemiological studies. Individual differences and external factors with impact on brain function: sleep, fatigue, nutritional factors, movement and exercise, risk factors and protecting factors for successful, normal or subnormal development, effects of training, emotional support and inspiration. Conditions: giftedness, developmental dysfunction, AD(H)D, autism and related disorders, learning problems, dyslexia, dyscalculia, language dysfunction, non-verbal learning disorder, stress, anxiety or mood dysfunction, addiction (alcohol, drugs), aggression.

### Toward a Curriculum on Neuroscience Education for Educators

The description of “the neuroscience issues that every educator should know” (see section “Four Key Issues About the Brain” and Box 2) is a proposal on content, not on approach. It is quite an undertaking to make a translation from key issues and core concepts (Box 2, Society for Neuroscience, 2008) such as described in the present paper into a curriculum. There is only a limited amount of scientific information available on the effects of application of neuroeducation on the educational practice or attitudes and approach of teachers. A recent review on the results of neuroscience training for teachers in Trends in Neuroscience and Education, TiNE; Privitera, 2021) found only ten papers in which the description of the neuroscience courses used was of sufficient detail and quality to enable a comprehensive evaluation of the current research on neuroscience training for teachers. The authors of the TiNE paper found most results to be “promising” although there were quite some differences in the nature of the courses given, their contents, length, approach and the relative time spent on the various issues. The paper therefore supports the notion put forward in the present review, namely that the field is in need of a clear knowledge base on the scientific insights that the field of education needs. With the present article and especially with our proposal in chapter 4, we hope to provide a starting point for discussion among professionals from the fields of neuroscience, cognitive and behavioral science and professionals from the applied field of education, notably teacher trainers.

A stumbling block as to the organization of practical courses on neuroeducation is that up till now, there is only limited access to scientific literature, tools and written sources such as books, and courses aimed at teachers and other educational professionals. Moreover, accessible sources on the basics of Mind, Brain and Education science with both scientific knowledge and recommendations for educational practice are still very limited. An additional problem is that many sources are not yet based upon evidence-based or evidence-informed interventions in the educational setting. Yet, valid literature about the structure and functioning of the brain and about neuropsychological development does exist. This type of information can be found in textbooks which are written for undergraduate students in psychology. Examples are books such as “Introduction into Biological Psychology” (e.g., Kalat, 2018) and introductory texts in Neuropsychology (e.g., Kolb and Whishaw, 2015) and Developmental Cognitive Neuroscience (e.g., Goswami, 2019 or Blakemore, 2018). The advantage of these books is that they do not delve deeper in brain mechanisms and structure than is needed for an understanding of cognition, affect and behavior in relation to brain function, and that they have been used successfully for many years in major universities around the globe. The use of these well-written books has an additional advantage in that there are many examples of existing courses which are based upon these books. This makes it easier to make a new course for (pre-service or in-service) teachers in which existing examples of successful courses can be used to decide upon the nature and the volume of the to-be-learnt material. These basic books can be complemented with more specialized information. See Box 3 with a list of easily accessible books on topics as reviewed in the present paper. One of the earlier accessible books was *The learning brain* by Blakemore and Frith (2005). These authors already stated that a shared vocabulary is needed between neuroscientists and educators. In the past decade, some books have appeared that do make a translation of neuroscience content or insights about Executive Functioning to the classroom. Recent books on the translation of neuroscience insights to the classroom are those by Tokuhama-Espinosa, 2014; Dawson and Guare, 2018; Hohnen et al., 2019). In addition to that, accessible books on the adolescent and his or her development are those by Steinberg (2014, 2019); these books provide important information on the adolescent and “the age of opportunity” with implications for pedagogical approach and attitude.

**Box 3. Textbooks on the learning brain and the developing child and adolescent.**

*The books and literature which are described in this box are easily accessible and are “suggested reading” for educational professionals who wish to increase their knowledge and insights into the developing child and adolescent, learning and cognition. The full reference with bibliographical details can be found in the reference list.*

Blakemore, S.-J., and Frith, U. (2005). *The learning brain. Lessons for education*.

Bjorklund, D. F. (2020). *Child Development in Evolutionary Perspective*.

Blakemore, S.-J. (2018). *Inventing ourselves: The secret life of the teenage brain*.

Dawson, P. and Guare, R. (2018). *Executive Skills in Children and Adolescents*.

Dehaene, S. (2020). *How We Learn: The New Science of Education and the Brain*.

Galvan, A. (2017). The Neuroscience of Adolescence.

Goswami, U., (2019). *Cognitive Development and Cognitive Neuroscience: the developing Brain*.

Gray, P., and Bjorklund, D.F. (2018). *Psychology*.

Hohnen, B., et al. (2019). *The incredible teenage brain: Everything you need to unlock your teen’s potential*

Jolles, J. (2016). *The teen brain. On the adolescent between biology and environment (in Dutch)*.

Jolles, J. (2020). *Learning to know your child. On development, learning, thinking and the brain (in Dutch).*

Kalat, J.W. (2018). *Biological Psychology*.

Kolb, B., and Whishaw, I.Q. (2015). *Fundamentals of Human Neuropsychology* 7th ed.

Steinberg, L. (2014). *Age of Opportunity. Lessons from the New Science of Adolescence*

Steinberg, L. (2019). *Adolescence*.

Thomas, M. S. C., et al. (2020). *Educational Neuroscience: Development Across the Lifespan.*

Tokuhama-Espinosa, T. (2011). *Mind, Brain, And Education Science: A Comprehensive Guide To The New Brain-Based Teaching.*

Tokuhama-Espinosa (2014). *Making Classrooms Better*.

### Essential for Educational Innovation: A Transdisciplinary Approach

It is imperative that curricula for educational professionals are developed from a multidisciplinary and multidimensional angle and that they are based upon a transdisciplinary attitude. Teacher trainers have an important role in that respect because they are specialists who have a vision about what the educational professional should know and why. They are also important to support teachers to become neuroscientifically literate as defined in the introduction of this article: they can help to further development of neuroscience literacy as a concept that demands for competence on reflective assessment of knowledge and to stimulate teachers to adopt a critical-reflective teaching method (e.g., Bergmann et al., 2017). Experts in the neurosciences and the cognitive and behavioral science contribute by proposing the relevant content from their domains, whereas specialists in neuropsychological development and cognitive performance are needed to contribute by giving directions about (sources of) individual differences, about factors contributing to the efficiency of information processing and about interventions that have proven effectiveness in the intervention of individuals with a cognitive dysfunction or a brain disorder.

In conclusion, what is needed is a translation and integration of knowledge that transcends the boundaries of the various domains, leading to a holistic or “transdisciplinary” approach to the study of learning and education. Transdisciplinary academic networks in which universities make formal collaborations with schools and institutes which are responsible for teacher training are useful in this respect. Such networks could stimulate the constructive dialog between disciplines and support individuals from various backgrounds to address educational innovations. International organizations such as the International Mind, Brain and Education Society (IMBES) and The European Association for Research on Learning and Instruction (EARLI) are vital for information exchange and collaborations on a higher level. In addition, a major role is to be played by specialists in science communication and experts in the use of the internet and social media. Special reports by international organizations like the DANA foundation, the Education Endowment Foundation, the Jacobs Foundation and the Society for Neuroscience and others could also play an important role.

## Concluding Remarks

The present article suggests that there is some lack of progress on the topic of neuroeducation which has to do with three major factors. In the first place, research in the past fifteen years has placed the emphasis on the results of experiments in which brain imaging methods (notably Magnetic Resonance Imaging, MRI) have been used. In retrospect, the neuroimaging experiments have yielded interesting scientific results, which have deepened our understanding of brain mechanisms underlying cognitive and affective processes. Yet, the fundamental and unidimensional nature of most imaging studies prevents a direct application to the field of education. Future research should take a transdisciplinary approach to take on problems and questions *from the field of education*, investigating the same issue on multiple levels of analysis. Thereby, neuroimaging research, laboratory studies with well-controlled behavioral tasks, and classroom studies could mutually inform and constrain one another. Still, at present, there is relevant knowledge about the learning brain, which appears to lie in an improved understanding of how to bring the brain in an optimal condition to learn, and by stimulating insight into external, non-psychological factors which act upon the learning individual. The vast amount of knowledge about “brain plasticity” and related topics yields predictions that could help to optimize the conditions for information processing and learning. Educational interventions in which sleep and fatigue, nutritional status, attentional processes or movement are manipulated are examples of approaches that may prove of value and deserve the attention of educational professionals. Nonetheless, we would like to re-emphasize that neuroscientific insights need to be combined with insights from other domains to form hypotheses about learning in the daily context. Educational researchers may play an important role in testing these hypotheses, enabling the conversion of true scientific insights into scalable practical applications.

In the second place, there is a major lack of valid sources of information (books, articles, courses, internet sources etc.) for use by the interested educator. The fact that neuromyths are still prevalent (Dekker et al., 2012; Howard-Jones, 2014; Macdonald et al., 2017) and the seductive allure of neuroscience as well as our expectations about the contributions of brain training techniques (too optimistic) underscore our plea for the development of a curriculum for educators which makes use of valid sources which can be trusted. We suggested to use existing handbooks and textbooks on the domain of biological psychology and neuropsychology that are already in use for university students in the behavioral sciences.

In the third place, there is a substantial confusion of tongues with respect to the potential importance of neuroscience and cognitive science knowledge and insights. This is evident in opinions that are expressed in statements such as “a bridge too far,” “beware of the brain hype” and visions stating that teachers do not need anything more than a good behavioral observation. To present an analogy with the applied field of health and disease: it is unthinkable that a medical practitioner or health care psychologist would have *no* knowledge about biology, about the structure and functioning of the heart, the digestive system, the brain and other organs and about the internal and external factors which determine functioning of the individual (see also Thomas, 2013). We are convinced that this also applies for the educational professional. Knowledge about the brain and its development and maturation, and about the factors which are responsible for normal, subnormal and successful learning can provide a context for a better understanding of behavior.

We feel that the criticisms related to the pretenses of cognitive neuroscience research are understandable. A statement arguing that “*we currently do not know enough about the brain to provide concrete recommendations for didactics and teaching*” is fair, as has been explained in this paper. It is indeed not possible to translate neuroscience insights directly into innovative didactics and educational interventions. However, apart from prescription about teaching, there is conceptual knowledge about the interplay between mind, brain and education. This knowledge is useful for teachers, as it could help them to contextualize children’s behavior, inspire them, and/or assist them in making educational decisions and support their pedagogy. Another negative opinion about the relevance of neuroscience is that “*teachers know best how to interact with their students and that they should not lose their autonomy*.” This notion is understandable because scientific research has provided general insights, which are not directly applicable to student A or student B. Notwithstanding that fact, the neuroscientific insights will hopefully give teachers more rather than less autonomy, as these insights could help them make more informed decisions (Dehaene, 2020), while staying true to their personal educational goals. Neuroscience is just one piece of the complicated puzzle of learning and education.

In sum, many of the remarks on the pretenses of the neurosciences and their possible impact for the field of education are understandable. They point to a confusion of tongues between disciplines. This implies that we should seek to stimulate the dialog and use a translational approach. In that respect, it is of importance to change the attitude of the various disciplines and participants and promote a mutual respect for the knowledge, insights and methods of other disciplines. This means: respect for the representatives from other disciplines and helping each other to acknowledge the existence of a language gap which can lead to stumbling blocks and lack of progress. We plead for a collaboration between the various fields, in analogy to the collaboration between the fundamental and applied disciplines in the multidimensional field “health and disease.” The implications are, that it is essential to come to a reorientation of knowing and knowledge, insights and science. We have to change our attitude and come to a multidimensional and multidisciplinary approach in educational innovation. Scientific insights into learning, about the learning brain and about factors that are responsible for normal, successful and suboptimal learning can help the educational professional to create the optimal conditions for talent development in his students.

## Author Contributions

Both authors listed have made a substantial, direct, and intellectual contribution to the work, and approved it for publication.

## Conflict of Interest

The authors declare that the research was conducted in the absence of any commercial or financial relationships that could be construed as a potential conflict of interest.

## Publisher’s Note

All claims expressed in this article are solely those of the authors and do not necessarily represent those of their affiliated organizations, or those of the publisher, the editors and the reviewers. Any product that may be evaluated in this article, or claim that may be made by its manufacturer, is not guaranteed or endorsed by the publisher.
